# A Randomized Controlled Trial on the Effects of Yoga on Stress Reactivity in 6th Grade Students

**DOI:** 10.1155/2013/607134

**Published:** 2013-01-30

**Authors:** Marshall Hagins, Sara C. Haden, Leslie A. Daly

**Affiliations:** Department of Physical Therapy, Long Island University, Brooklyn Campus, One University Plaza, Brooklyn, NY 11201, USA

## Abstract

There is an increasing interest in developing school programs that improve the ability of children to cope with psychosocial stress. Yoga may be an appropriate intervention as it has demonstrated improvements in the ability of children to manage psychosocial stress. Yoga is thought to improve the control of reactivity to stress via the regulation of the autonomic nervous system. The current study examined the effects of yoga compared to a physical education class on physiological response (blood pressure (BP) and heart rate (HR)) to behavioral stressor tasks (mental arithmetic and mirror tracing tasks). Data analysis of BP and HR was performed using a 2 × 2 × 4 repeated measures ANOVA (time × group × stressor time points). 30 (17 male) 6th graders participated in the study. Yoga did not provide significant differences in stress reactivity compared to a physical education class (group × time: systolic (*F*(1,28) = .538, *P* = .470); diastolic (*F*(1,28) = .1.061, *P* = .312); HR (*F*(1,28) = .401, *P* = .532)). The lack of significant differences may be due to the yoga intervention failing to focus on stress management and/or the stressor tasks not adequately capturing attenuation of stressor response.

## 1. Introduction

Excessive or prolonged psychosocial stress in children and adolescents has been shown to be associated with pathology [1–3] and negative school behaviors [4–7]. Consequently, there is increasing interest in developing school programs that may improve the ability of children to be resilient and cope with psychosocial stress [8]. Yoga may be an appropriate intervention to reduce stress. There is no single definition of the practice of yoga that is universally accepted although it is generally described as an ancient tradition [9–11] that uses techniques of posture (asana), breath control (pranayama), and meditation, as well as moral and ethical observances [12, 13]. A more complete description of the historical origins of yoga and its philosophy can be found elsewhere [10, 14]. Reviews of the current literature suggest that yoga can reduce stress and stress-related dysfunction in adults [15–17], although most of these reviews suggest caution is warranted as the majority of studies have substantial methodological limitations. A much smaller number of studies have examined the effect of yoga on stress in children. In general, these studies have shown that yoga improves the ability of children to manage psychosocial stressors [18, 19]. Specifically, controlled studies on children have demonstrated improvement with the practice of yoga on anger [20, 21], depression [20], stress [22], negative affect [22, 23], body dissatisfaction [24], self-concept [24], anxiety [25], and positive affect [25]. 

The mechanism by which yoga may improve the ability to deal with psychosocial stress remains unclear. Current theory suggests that yoga alters the extent to which events are experienced as stressful or impacts the reactions to perceived stress [26]. This theory suggests that the degree to which yoga improves stress management is directly linked to regulation of autonomic arousal. Therefore, measures such as blood pressure (BP), heart rate (HR), and heart rate variability (HRV) may provide additional sources of information to understand both the efficacy and the mechanisms of yoga. 

A common experimental method to determine autonomic arousal to stressful stimuli is to introduce a stressful behavioral task (e.g., math test, social presentation task) and measure the degree of physiological response or *stress reactivity*. In adolescents, stress reactivity has been shown to be related to hypertension [27, 28] and atherosclerosis [29, 30] suggesting that repeated stress reactivity may be involved in the pathogenesis of cardiovascular disease [31]. In children, stress reactivity has been shown to be related to future blood pressure status [32] as well as to levels of adiposity [33]. Stress reactivity in children has also been associated with negative behaviors related to the ability to navigate stressful situations and regulate emotions [34, 35]. Laboratory methods used to measure stress reactivity in children have been shown to successfully approximate individual differences in reactivity to typical daily stressors [36–38].

To our knowledge only five studies have specifically focused on the effects of yoga in collaboration with a school system [21, 23, 39–41] and none have used physiological measures related to stressful events. The earliest paper [41] was written without fundamental details such as descriptions of the measures and nature of the findings (i.e., within or between-group effects). The remaining four papers rely solely on self-report questionnaires on psychosocial variables (with the exception of one study [39] which also examined hamstring flexibility and one leg balance). Although self-report measures on psychosocial variables are relevant to health and educational outcomes, they may be influenced by social desirability and other sources of bias and should be balanced with other relevant measures. In particular, given the prevailing theory regarding mechanisms underlying effects of yoga as regards to the experience or perception of stress, it would be useful to measure physiological values thought to represent autonomic arousal, namely, BP and HR. Therefore our purpose was to test the hypothesis that a yoga program would provide a significant reduction in stress reactivity in 6th grade students compared to that provided by a standard physical education class. The secondary purpose was to explore the feasibility of implementing behavioral stressors in a study within a busy New York City (NYC) public school. 

## 2. Materials and Methods

The current study was a pilot randomized controlled trial of 6th grade students assigned to either a 15-week yoga (Y) or physical education (PE) program using pre- and posttest measures. HR and BP were obtained relative to the introduction of behavioral stressors. Psychological measures were obtained including self-report measures on affect, proactive and reactive aggression and self-worth, as well as parent reports on emotional and behavioral problems. Results from the psychological measures will be reported elsewhere.

Prior to recruitment, this study was approved by both the NYC Department of Education and the Long Island University Institutional Review Boards. Parents of all 6th grade students entering a public middle school in NYC were informed of the study via letters, emails, and a brief presentation by the primary investigators during fall semester student orientation. The students were informed of the study by the regular PE class instructor and the primary investigators within the first week of the semester. The inclusion criteria were students currently attending middle school in good general health as evidenced by permission to attend physical education class. After receiving a consent form from the parents and an assent form from the students, the eligible students were enrolled in the study and randomly assigned to either a 15-week PE or yoga course. 

## 3. Measures

Measures of recruitment and attendance were obtained by both PE and yoga instructors. As significant differences in active engagement with the intervention between groups might bias findings, a “child engagement index” was created. The respective PE or yoga teacher completed this index for each child at two time points within the trial period (approximately 5 and 10 weeks into the trial). The scale was a 3-point index anchored by the terms “minimal, moderate, and maximum engagement,” with narrative text describing each term (see Supplementary Appendix 1 in Supplementary Material available online at http://dx.doi.org/10.1155/2013/607134). 

Two behavioral stressors were identified that had been previously used successfully in studies of stress reactivity in children: the mental arithmetic task (MAT) [42–44] and the mirror tracing task (MTT) [45, 46]. The MAT asked the participant to subtract 23 from 1021 as quickly and accurately as possible within a 3-minute period. If the participant made a mistake he/she was asked to start all over again at 1021. In the MTT participants were asked to use a metal stylus to trace a star using only a mirror version of the star for guidance. The reversal of the image in the mirror makes tracing the star difficult. When errors occur by going outside the margins of the star design a buzzer sounds and each error is tracked on an electronic counter in full view of the participant and researcher. The participant is told to trace the star as many times as possible without any errors during a 3-minute period. Two types of stressors were used within this study in order to provide preliminary results regarding effects of stressor type (MAT versus MTT) on the physiological variables. The administration order of tasks was counterbalanced. Systolic and diastolic BP, as well as HR, were obtained through an automated blood pressure cuff (Omron: HEM-705CP; CT, USA) shown to have good reliability and validity across multiple studies [47–49]. 

Parents completed a demographic survey consisting of information about the child's primary parental figures, the nature of those parental figures (i.e., biological, adoptive-, step, or foster parent), employment status, the family income, and any medical issues that the child may have. 

## 4. Interventions

Yoga and PE classes occurred for the same duration (approximately 50 minutes each session) and frequency (three times per week) and at the same time of day throughout the study. The yoga intervention was provided by instructors trained in the use of a written yoga curriculum in compliance with New York State Standards for Physical Education. The two teachers providing the yoga instruction had been teaching children using this curriculum for 5 and 3 years, respectively. Both teachers had achieved a 200-hour yoga certification and then received 100 hours of training specific to the yoga curriculum provided within this study. 

The class consisted of (1) an opening ritual (centering, conscious breathing); (2) 30-minute asana practice (standing, seated, backbends/inversions) with each pose held for a 5 count, or occasionally taught as a “vinyasa flow” linking all the poses together for one breath; (3) brief seated meditation; (4) closing ritual of guided relaxation in Savasana (body scan). Homework on a specific aspect of the practice was encouraged each week. All classes integrated the 8 limbs of yoga but in an indirect and varied manner (no required memorization of Sanskrit terms). See Supplementary Appendix 2 for more details. 

The PE class used common games such as soccer and volleyball as well as an indoor walking program to encourage moderate levels of physical activity and to provide an opportunity for social interaction among students. Approximately 75% of the total time was spent in physical activity of games or walking, while approximately 25% was spent being physically inactive during attendance and instruction on rules of the various games or related instruction.

## 5. Procedures 

The procedures and measures in pre- and posttests were identical. Pretesting occurred one to two weeks before the start of the intervention (late September), and the posttesting occurred one to two weeks after the final class (early January). Two of the three primary investigators and five trained evaluators performed all measures. The remaining single primary investigator was not blinded to group assignment and did not participate in measures in order to implement necessary administrative record keeping and collaboration with the school. 

Participants were measured during the time periods normally used for PE class, advisory class, or lunch in order to minimize disruption to their education, as was required by the school administration. Two participants at a time were tested in a small room (PE instructor's office). One of the two evaluators flipped a coin to determine which stressor one of the participants would be administered first. The second participant automatically started with the other stressor. This was done in order to prevent both participants from performing the same stressor at the same time within the confined space where measurements were being performed, thereby minimizing interaction between the two participants. This procedure also effectively randomized and counterbalanced the behavioral stressors. The participants were seated and the procedures were explained and their questions answered. Evaluators determined if the participant had ingested caffeine or exercised previously that day and, if so, the participant was asked to return another day for testing. The cuff for the blood pressure device was placed on the nondominant arm using the appropriate size cuff while keeping the arm horizontal at heart level and placed on a table. The cuff was inflated to determine a comfort level for the participant and ensure successful operation. Throughout the session evaluators read the electronic output of the automated BP monitor to obtain BP and HR data and recorded the data on standardized worksheets. 

After the placement of the cuff, the evaluator provided the participant with a magazine and asked him/her to remain at rest and relax for five minutes. At the end of the five minutes two successive initial resting BP and HR measurements were taken. The evaluator read a standardized script that briefly described the behavioral stressor that the participant was to begin. Both stressor tasks (MAT and MTT) were each three minutes long. A one minute break in-between the two stressor tasks was inserted to allow the evaluator to repeat standard directions for the second stressor task. Throughout both stressor tasks the evaluators maintained a serious demeanor and emphasized the importance to the participant of making their best effort. At the halfway point and at the end of each stressor task, 1.5 and 3 minutes, BP and HR measures were taken. At the end of the second stressor the participant was once more given a magazine and asked to relax for 10 minutes. Recovery BP and HR values were obtained at the end of 5 and 10 minutes. Therefore, in total, BP and HR data were collected at 8 time points across the entire session (2 initial rest values, 2 values for each of the two stressors, and 2 recovery rest values). The BP and HR device was then removed from the participants and they were given food (e.g., pizza) which served as an incentive for participation and also as lunch for those who were measured during their lunch period. 

After the pretest a graduate assistant randomly assigned each student to either the yoga or PE intervention by drawing names from a hat assigning each subsequent name to a different intervention. The randomization was conveyed to the primary yoga and PE teachers via concealed envelopes. The primary investigators and all evaluators performing measurements remained blinded to group assignment. Given the active interventions, participants were necessarily aware of group assignment. All 6th grade students were required to assemble in a single group within the school's gymnasium each day prior to the interventions and then those in the yoga group moved to a separate classroom for yoga instruction. Posttest measurements were implemented in an identical manner at the end of the interventions.

## 6. Data Analysis

The current paper focuses only on group effects related to blood pressure and heart rate and issue of feasibility specifically related to methods using behavioral stressors. The two initial resting and two recovery rest values were each averaged to create one mean value representing the initial and recovery values for BP and for HR. Paired *t*-tests demonstrated that BP and HR values taken at the middle of each stressor (1.5 minutes) were not significantly different than these at the end point (3 minutes) of each stressor (all *P* < .05). Consequently, to simplify and strengthen the statistical approach, only the values at the end of each stressor were used. Therefore, four final values were used for analysis of each session ((1) mean initial rest, (2) end of stressor 1, (3) end of stressor 2, (4) mean recovery rest). 

Data were analyzed using SPSS (Version 13). Baseline equivalence of the PE and yoga groups relative to age, gender, ethnicity, annual family income, medication, attendance, and engagement index were determined using independent *t*-tests or chi squared tests as appropriate. BP (systolic and diastolic) and HR values at pre- and posttest were evaluated for normality for parametric assumptions and were evaluated for baseline equivalence between groups at pretest using independent *t*-tests. As a pilot study exploring optimal methods of implementing behavioral stressors to 6th grade students, our analysis also focused on the ability of the stressors to successfully create significant physiological stress reactivity. Consequently, this question was included in the model and the BP and HR values were examined with a 2 × 2 × 4 repeated measures ANOVA (time × group × stressor time points). Additionally, we were concerned with differential effects of the two types of stressors. Due to the counterbalanced design, the first stressor for half the participants was the MAT and for the other half it was the MTT. Due to potential confounding effects of carryover from the first stressor to the second stressor comparisons were only made on the stressors given first. Therefore, in a separate analysis the effect of type of stressors was compared using independent *t*-tests. 

## 7. Results 

41 consent forms were received back from the parents of the 61 eligible students (67.2% return rate). One returned consent form was discovered to be for a child in a different grade, nine children did not assent to the study, and one child decided not to continue in the study for the posttest measures. Consequently, the final number of participants was 30 (49.1% of potential subjects) and, of those, 24 of the parent demographic forms were returned. 

Initially 16 subjects were assigned to PE and 15 were assigned to yoga. However, as noted, one child dropped out of the study after the pretest resulting in 15 subjects assigned to each condition. Based on available demographic information on 24 out of the 30 subjects, the sample consisted of 56.7% males and the mean age was 10.8 years (SD = .41, range 10-11 years old), 50% of the students were Caucasian, 16.7% Hispanic, 8.3% Asian, 4.2% Black, and 20.8% reported other races. 

Baseline analyses showed no significant differences between groups in demographic variables, BP, HR, attendance, or engagement with the intervention (Table 1). Blood pressure and HR distributions met assumptions for parametric analysis. The independent *t*-tests comparing the first stressors (MAT versus MTT) used during the pretest found that there were no significant differences in BP or HR values relative to the type of stressor used (systolic: *P* = .999; diastolic: *P* = .760; heart rate: *P* = .251). 

Means and standard deviations of BP and HR at the four time points for both pre- and posttest are displayed in Table 2. Mauchly's test of sphericity indicated that the assumption of sphericity had been violated, and therefore a Greenhouse-Geisser correction was used for all results. Results of the 2 × 2 × 4 repeated measures ANOVA found no significant three-way or two-way interactions on any physiological variable. The values for the primary outcome of interest (group × time) were systolic (*F*(1,28) = .538, *P* = .470); diastolic (*F*(1,28) = .1.061, *P* = .312); HR (*F*(1,28) = .401, *P* = .532). This finding suggests that there were no effects on any physiological variable relative to group. There were significant main effects due to time (pre- to posttest) for systolic and diastolic values but not for HR: systolic (*F*(1,28) = 6.206, *P* = .019); diastolic (*F*(1,28) = 8.435, *P* = .007); HR, (*F*(1,28) = .820, *P* = .373). This finding suggests that BP, but not HR, increased significantly from pre- to the posttest when viewed across both groups and stressor time points. There were also significant main effects across the four measurements taken within a single testing session (initial rest period, end of stressor 1, end of stressor 2, recovery period) for BP and HR: systolic (*F*(2.51,70.20) = 11.61, *P* < .0005); diastolic (*F*(2.24,62.72) = 12.44, *P* < .0005); HR, (*F*(2.37,66.47) = .10.22, *P* < .0005). For all variables there were significant increases in BP and HR values from initial rest compared to the end of each stressor. Consequently, the finding for stressor time points viewed across group and time for all three physiological variables suggests that the stressors successfully engaged a stress reactivity response from the participants. 

## 8. Discussion

This study found that 15-week yoga program did not provide significant differences in stress reactivity compared to a physical education class in 6th grade students. As this was a randomized study with blinded investigators and the demographic variables, attendance, and engagement index values were equivalent across groups, sources of potential bias were minimized. Consequently, these findings are likely to represent a true failure of the specific yoga program within this study to provide significant reductions in stress reactivity. The results do not support suggestions that benefits from yoga are derived from a mechanism related to increased regulation of the autonomic nervous system. 

To our knowledge, no studies have examined the effects of yoga on physiological stress reactivity in children, making direct comparisons difficult. However, a limited number of studies have shown various interventions to significantly decrease stress reactivity. Adults using transcendental meditation [50, 51]), biofeedback [52, 53], and children using exercise [54] or a walking program [55] have shown significantly reduced stress reactivity after intervention. The mechanism underlying the significant change in stress reactivity in these studies remains unclear but is likely specific to each intervention method. Meditation interventions have been suggested to reduce sympathetic nervous arousal [56, 57], while aerobic fitness has been shown to be inversely related to stress reactivity [58, 59] and may alter stress reactivity by decreasing vascular resistance and increasing parasympathetic tone. Studies using biofeedback suggest that improving subjects' ability to differentiate internal cardiovascular sensations allows a greater degree of control of that system relative to emotional life events [52]. Similarly, the expectation underlying the current study's hypothesis for an effect of yoga on stress reactivity was that yoga would alter the extent to which events are experienced as stressful or the reactions to perceived stress. 

The negative results in the current study may be related to the specific manner in which the yoga practice was implemented and its failure to directly address the issue of reaction to perceived stress. Although the intervention within the current study used a frequency and duration (15 weeks, 2 times per week for one hour each) similar to existing studies of yoga on children in a school setting [21, 23, 39, 40], the relative importance of these factors to outcomes such as stress reactivity is unknown. Perhaps more important, the yoga program did not focus on reducing the perception or experience of psychosocial stress, although that was the primary outcome of interest and the underlying theory being examined. It is possible that a yoga program which had more extensive focus on explicit training on the initial awareness of and conscious coping mechanisms related to stressful events may provide different outcomes in terms of stress reactivity. 

An additional factor, unexpected and undesired, arose during implementation of the yoga practice which may have influenced the negative results. Some students randomized to the yoga program begin to express a negative attitude about participating in yoga because they were “jealous” of their peers who were spending the same time periods playing soccer, basketball, and various games. Despite the investigators' desire to provide students with an exciting, pleasurable, and interactive yoga intervention, all students did not perceive it that way. The degree to which these perceptions influenced the outcomes is unclear. However, future studies may benefit from consideration during the design stage regarding the mechanics of randomization and controlling for participant expectations. 

A second factor potentially relevant to the negative results is the type of stressor task used within this study. In general, the mechanisms relating type of stressor task to degree of potential for attenuation of stressor reactivity remain poorly understood. Importantly, the positive findings in the previously cited studies above all occurred with the use of stressor tasks different from those in the current study (e.g., stroop task, public speaking, simulated driving, etc.). Other studies that used an MTT and/or the MAT (as in the current study) did not find significant changes due to the intervention [60] or had varying results relative to the type of stressor task within the study [53]. The stressor tasks in the current study were chosen primarily for practical reasons (requiring little time to complete, limited space and supervision) and, for the MAT, because it closely simulated a stressor which children experienced during school. It is possible that other stressor tasks would have provided different results relative to a yoga intervention. For example, it has been suggested that more socially relevant tasks produce greater and more consistent stress reactivity [61] and, consequently, stressor tasks such as a speech task may be more appropriate for this population.

The secondary purpose of this study was to explore general features of feasibility related to the use of behavioral stressors. The MAT and MTT used in this study successfully created stress reactivity in the students. Further, there were no significant differences in the effects generated by the two stressors (MAT and MTT). Based on these results alone one could suggest that either of these stressors is appropriate for stress reactivity studies in children, and there may be little need to include both within one study design. However, as discussed above, negative findings in previous studies [53, 60] and the current study using these stressor tasks suggest that future studies on yoga should explore additional alternative stressor tasks. 

An interesting and unexpected finding was the significant increase in systolic and diastolic blood pressure at posttest when viewed across groups and stressor time points. Most studies of stress reactivity find a decrease in response to the same stressor over time. Why blood pressure values would rise when viewed across group and stressor time points during the posttest is unclear although there is some evidence for increased stress reactivity when repeating the MAT stressor over a period of 10 days [62]. We speculate that this significant increase in reactivity may have been due to awareness of the nature of the stressor tasks during the posttest measurement. We cannot exclude other explanations such as increased psychosocial stress that may occur during the first year of middle school. 

This study was limited by a small sample size, and as such we did not include analysis relative to gender which may have provided interesting and unique results. For practical reasons of limited school space, the current study had to perform all measurements in a small room without adequate controls for noise or temperature and which allowed for the two study subjects to hear each other's responses, potentially influencing their own responses. These characteristics of the measurement procedures may have affected the results. 

## 9. Conclusions

This RCT of 6th grade students within a busy NYC public school found no significant differences in stress reactivity when comparing a 15-week intervention of yoga and physical education class. The nonsignificant results may be explained by the adequacy of the intervention relative to a lack of specific focus on training students regarding the awareness of and conscious coping mechanisms related to stressful events. Future yoga studies in this population may consider designing a yoga intervention that successfully competes with comparison groups for students' attention and desirability and which specifically and explicitly focuses on the student's approach to stressful events. 

## Supplementary Material

Appendix 1: Engagement Index.
Appendix 2: Description of the yoga class.

## Figures and Tables

**Table 1:** Demographics and baseline equivalence.

Characteristic	Yoga group		PE group		Statistics	
Age (*N* = 24)	*M* = 10.75 years old (SD = .45)		*M* = 10.83 years old (SD = .39)		*P* = .63	

Gender (*N* = 30)	Male = 10		Male = 7	
	Female = 5		Female = 8		*P* = .46	

Ethnicity (*N* = 24)	Caucasian = 7		Caucasian = 5	
	Black = 0		Black = 1	
	Asian = 1		Asian = 1		*P* = .82	
	Hispanic = 2		Hispanic = 2	
	Other = 2		Other = 3	

Annual income (*N* = 22)	$50,000 or below = 4		$50,000 or below = 0	
	$51,000−$100,00 = 2		$51,000−$100,000 = 5		*P* = .16	
	$101,000−$200,000 =2		$101,000 −$200,000 = 4	
	$201,000 and above = 3		$201,000 and above = 2	

Take medication (*N* = 20)	No = 10		No = 9		*P* = .31	
	Yes = 0		Yes = 1	

Attendance (*N* = 30)	*M* = 26.87 classes (SD = 4.85)		*M* = 22.80 classes (SD = 7.36)		*P* = .09	

Systolic BP (*N* = 30)	99.67 (18.01)		101.07 (12.78)		*P* = .808	

Diastolic BP (*N* = 30)	62.73 (10.39)		64.93 (9.24)		*P* = .545	

Heart rate (*N* = 30)	84.23 (8.07)		85.06 (13.50)		*P* = .839	

Engagement scales (time 1,2: *N* = 28,26)	*Time 1 *	*Time 2 *	*Time 1 *	*Time 2 *	*Time 1 *	*t *
Minimum	0	0	0	0		
Moderate	4	5	8	5	*P* = .13	*P* = 1.00
Maximum	10	8	6	8		

*M*: mean; SD: standard deviation; BP: blood pressure; units for BP: mmHg.

**Table 2:** Means and standard deviation of systolic, diastolic, and heart rate.

		Pretest								Posttest							
		Initial rest	SD	Stressor 1	SD	Stressor 2	SD	Recovery rest	SD	Initial rest	SD	Stressor 1	SD	Stressor 2	SD	Recovery rest	SD
Systolic	Yoga	103.8	17.6	106.0	17.6	106.3	16.7	101.2	13.5	104.5	11.9	116.2	12.7	108.6	10.7	112.2	12.9
	PE	101.6	13.5	111.4	13.5	108.9	11.9	107.0	11.1	106.7	11.8	112.3	12.1	115.2	13.4	105.1	12.3

Diastolic	Yoga	64.3	10.8	67.7	9.1	69.0	11.7	62.8	7.5	64.0	9.4	76.8	9.9	70.2	6.7	68.6	8.5
	PE	65.9	8.8	76.9	14.0	69.4	7.9	72.2	11.6	69.8	11.0	76.6	11.5	75.3	8.6	72.1	10.3

Heart rate	Yoga	83.2	8.1	87.4	12.5	93.1	13.7	84.5	10.8	85.1	13.1	89.8	15.1	92.8	12.6	86.5	9.7
	PE	85.1	14.0	95.4	20.0	92.6	18.0	86.9	14.5	87.9	13.9	92.3	15.3	94.9	18.4	90.7	12.6

Note: initial rest: mean of first two measurements after 5-minute rest. Stressors 1 and 2: values taken at the end of 3 minute stressor tasks 1 and 2. Recovery rest: mean of final two measurements taken at 5 and 10 minutes after the end of second stressor. SD: standard deviation.
