# Supplementary material for: A Randomized Controlled Trial on the Effects of Yoga on Stress Reactivity in 6th Grade Students
Source: Evid Based Complement Alternat Med. 2013 Jan 30;2013:607134. doi: 10.1155/2013/607134 (PMC3572691; doi:10.1155/2013/607134)
Supplement: Supplementary file 1 — Appendix 1: Engagement Index. Appendix 2: Description of the yoga class. [file 607134.f1.docx]

**Appendix 1. Engagement Index**

1- Minimal engagement- child appears distracted and not engaged in the session, talks and/or disrupts the class and/or other children more than 2 times within the observation period; body posture and movements suggest a lack of motivation to participate in the activities
2-Moderate engagement- child appears engaged or somewhat engaged in the session, talks and/or disrupts the class and/or other children 1 or 2 times within the observation period; body posture and movements suggest some motivation to participate in the activities 
3-Maximum engagement- child appears fully engaged in the session, does not disrupt the class or other children; body posture and movements suggest motivation to participate fully in all activities.

**Appendix 2: Description of the yoga class**

The class consisted of : 1) an opening ritual (centering, conscious breathing); 2) 30 minute asana practice (standing, seated, backbends/inversions) with each pose held for a 5 count, or occasionally taught as a vinyasa flow linking all the poses together for one breath; 3) brief seated meditation; 4) closing ritual of guided relaxation in Savasana (body scan). Homework on a specific aspect of the practice was encouraged each week. All classes integrated the 8 limbs of yoga in an indirect and varied manner (no required memorization of sanskrit terms).

| Opening ritual: Students sit in Sucasana or Virasana and perform centering and conscious breathing (Ujayii or Tri Par Veloma) | 3-7 mins. |
| --- | --- |
| Standing poses: (5-6 of the below each class) using the breath (Ujayi)  Surya Namaskar A (5x), Padangustasana, Trikonasana, Parsvokonasana, Virabhadrasana 1 &2, Vrksasana, | 15 mins. |
| Seated poses : (2-3 of the below each class)  Dandasana, Paschimotanasana, Janusirsasana A, Ardha matsyendrasana, Navasana, Poorsvotanasana | 5 mins. |
| Backbends and inversions: (1 of the below, repeat 3x)  Bridge pose with an option to do Urdhva dhanurasana, Dhanurasana, Salambasana, Sarvangasana, Halasana, L shaped handstand at the wall, legs up the wall. | 5-10 mins. |
| Seated meditation: both hands to the heart and silently sent gratitude to themselves and others. | 2-5 mins. |
| Closing ritual: Savasana with guided relaxation of body scan: “relax your toes, your toes are completely relaxed… scanning up the body until the mind is also relaxed.” Students lie silently for as long as they can. | 4 mins. |
| Homework was encouraged: assignments varied and included: pick one pose to practice once a day for a week, practice Sury Namaskar A every morning, put together your own sequence of 5 poses in a vinyasa style, practice the pose you dislike the most once a day, try to invent a new yoga pose and try to teach someone at home what you learned in class | |
| The eight limbs of yoga were instructed indirectly and in a varied manner across the entire program. For example during seated meditation students were asked to give gratitude to themselves, the people around them and the environment for allowing us a peaceful place to practice yoga (Ahimsa). In Savasana students were encouraged to practice Pratyahara by instructing them to let go of any distractions. One pointed concentration was encouraged in multiple ways such as using Drishti in a challenging balance pose, or focusing on counting the breath down from 10 to 1 in a seated meditation. | |
